# Characterization of dysphagia and laryngeal findings in COVID-19 patients treated in the ICU—An observational clinical study

**DOI:** 10.1371/journal.pone.0252347

**Published:** 2021-06-04

**Authors:** Hanna Osbeck Sandblom, Hans Dotevall, Kristina Svennerholm, Lisa Tuomi, Caterina Finizia

**Affiliations:** 1 Department of Otorhinolaryngology, Head and Neck Surgery, Region Västra Götaland, Sahlgrenska University Hospital, Gothenburg, Sweden; 2 Department of Otorhinolaryngology, Head and Neck Surgery, Institute of Clinical Sciences, Sahlgrenska Academy, University of Gothenburg, Gothenburg, Sweden; 3 Department of Anaesthesiology and Intensive Care Medicine, Region Västra Götaland, Sahlgrenska University Hospital, Gothenburg, Sweden; 4 Department of Anesthesiology and Intensive Care Medicine, Institute of Clinical Sciences, Sahlgrenska Academy, University of Gothenburg, Gothenburg, Sweden; University Hospital Eriangen at Friedrich-Alexander-University Erlangen-Numberg, GERMANY

## Abstract

**Background:**

Dysphagia appears to be common in patients with severe COVID-19. Information about the characteristics of dysphagia and laryngeal findings in COVID-19 patients treated in the intensive care unit (ICU) is still limited.

**Objectives:**

The aim of this study was to evaluate oropharyngeal swallowing function and laryngeal appearance and function in patients with severe COVID-19.

**Method:**

A series of 25 ICU patients with COVID-19 and signs of dysphagia were examined with fiberendoscopic evaluation of swallowing (FEES) during the latter stage of ICU care or after discharge from the ICU. Swallowing function and laryngeal findings were assessed with standard rating scales from video recordings.

**Results:**

Pooling of secretions was found in 92% of patients. Eleven patients (44%) showed signs of silent aspiration to the trachea on at least one occasion. All patients showed residue after swallowing to some degree both in the vallecula and hypopharynx. Seventy-six percent of patients had impaired vocal cord movement. Erythema of the vocal folds was found in 60% of patients and edema in the arytenoid region in 60%.

**Conclusion:**

Impairment of oropharyngeal swallowing function and abnormal laryngeal findings were common in this series of patients with severe COVID-19 treated in the ICU. To avoid complications related to dysphagia in this patient group, it seems to be of great importance to evaluate the swallowing function as a standard procedure, preferably at an early stage, before initiation of oral intake. Fiberendoscopic evaluation of swallowing is preferred due to the high incidence of pooling of secretion in the hypopharynx, silent aspiration, and residuals. Further studies of the impact on swallowing function in short- and long-term in patients with COVID-19 are warranted.

## Introduction

In March 2020, the World Health Organization declared the novel coronavirus disease (COVID-19) caused by the severe acute respiratory syndrome coronavirus-2 (SARS-CoV-2) a pandemic health emergency. Until January 2021 about 100 million cases have been confirmed worldwide with more than 2.2 million fatal cases [1]. About 567,000 confirmed cases of COVID-19 infection were reported in Sweden at the end of January 2021, including approximately 11,500 fatal cases and 4,800 cases treated in intensive care [2]. The first wave of patients admitted to intensive care and deaths occurred from the second half of March to the second half of June 2020. A subsequent increase of COVID-19 cases has emerged from the end of October 2020 and persists at the end of January 2021.

The current evidence indicates that a substantial portion of patients with COVID-19 develop dysphagia during the course of the disease [3–5]. In particular, dysphagia appears frequent among patients admitted to intensive care due to COVID-19 infection [6]. It has been hypothesized that the SARS-CoV-2 virus may induce injury in the central and peripheral nervous system [7] and that this may have an impact on sensory and motor functions related to swallowing [8, 9]. Furthermore, critical illness as a consequence of prolonged intensive care, intubation, tracheostomy, and respiratory failure may also impose negative effects on deglutition and laryngeal function in this patient group [8, 10]. Previous studies on post-extubation dysphagia in the ICU varies greatly between 3 and 84% depending on the methodology [11, 12]. A study on mechanically ventilated patients in the ICU with no prior history of dysphagia performed a fiberendoscopic evaluation of swallowing (FEES) post extubation demonstrated the presence of penetration and aspiration in 36% and 22% of patients, respectively [13].

To the author’s knowledge, there are no studies of objectively assessed dysphagia and laryngeal appearance in patients treated in intensive care due to COVID-19. The aim of this study was to describe the characteristics of oropharyngeal dysphagia and laryngeal function in a series of COVID-19 patients treated in the intensive care unit (ICU), through assessment with fiberendoscopic evaluation of swallowing (FEES).

## Materials and methods

### Patients

A prospective series of 25 patients with polymerase chain reaction (PCR) verified COVID-19 treated in the ICUs at Sahlgrenska University Hospital, Gothenburg, Sweden, were included in the study. The study group consisted of 23 males and 2 females with a mean age of 63 years (range 43 to 78 years). Patients were referred on initiative of the ICU/ward staff for assessment of swallowing function during or shortly after the stay at the ICU. The major qualifications for examination were that the patient was completely awake, able to sit up in the bed, able to cooperate, and able to manage respiration in intervals without ventilatory support during weaning.

All patients in the cohort required invasive mechanical ventilation for several days during their ICU stay (mean 25 days, range 5 to 55 days). During mechanical ventilation lung-protective ventilation strategy was applied [14]. Prone positioning was applied in 13 patients (52%) when the partial pressure of oxygen/fraction of inspired oxygen (PaO2/FiO2) was equal to or below 150 mm Hg with FiO2 equal to or above 60%. In this cohort, patients were sedated with propofol and an opioid infusion. Dexmedetomidine or clonidine were added to the regime in the majority of cases because of high sedation requirements. In some patients, midazolam was also added to provide adequate sedation. Muscle relaxants were given in bolus doses or infusion in patients with refractory respiratory dyssynchrony.

### Assessment

The assessment of swallowing function was performed by one of two speech language pathologists (SLP) using FEES. Both SLPs had extensive experience in dysphagia management, including dysphagia in tracheotomized patients, and were certified according the European Society for Swallowing Disorders FEES accreditation program [15].

The FEES was performed using a Xion EV-NC videofiberendoscope attached to a Xion Endoportable CFT-003 mobile workstation (Xion GmbH). The examinations were recorded on video. Prior to the endoscopy in all patients, the nasal mucosa of the most patent nostril was decongested and anesthetized locally with a Lidocaine 3.4%/Naphazoline 0.02% solution using cotton attached to a thin feeding catheter (Unomedical Purifeed, CH 06), in order to reduce discomfort. Care was taken not to anesthetize the pharyngeal mucosa.

Personal protective equipment was used by the examiner and other staff in the room during the FEES, according to local hospital routine, in order to protect from possible decontamination by aerosol (N95/FFP3 face mask/respirator, face shield, hair protection, covering gown, and gloves). No transmission of COVID-19 occurred due to the FEES examinations. The fiberscopes were cleaned and disinfected after each examination using an automated endoscope reprocessor (dishwasher) as recommended by the manufacturer.

Boluses with different consistencies and volumes colored with green coloring agent were presented using a standard protocol; carbonated water (corresponding to International Dysphagia Diet Standardisation Initiative, IDDSI level 0 [16]), thin non-carbonated liquid (IDDSI 0), mildly thick liquid (IDDSI level 2), and extremely thick liquid/puree (IDDSI level 4). In most cases, 3–5 ml of carbonated water was given first, followed by increasing volumes of a mildly thick liquid, extremely thick liquid/puree, and lastly thin liquid without and with carbonation. The choice of boluses was made according to the clinical status and function of the patient, i.e., in cases with more severely impaired swallowing function specific boluses that were considered unsafe or not necessary for the clinical assessment were avoided.

Sixteen of the 25 patients were assessed with FEES on two or more occasions. Nine patients had two FEES, five patients had three FEES, and two patients were assessed with FEES four and five times, respectively. The median time to the first FEES was 35 days after admission to the hospital (mean 40 days; range 13 to 82 days). The first FEES was performed in the ICU in ten patients and in 15 patients after discharge from the ICU. Twenty patients were tracheotomized. All FEES examinations were performed with the patients off the ventilator.

In five patients with tracheostomy the examinations were performed during weaning from mechanical ventilation. None of the patients with tracheostomy had inflated tracheal cuff during the swallowing examination.

Data of the total time in hospital, time in the ICU, time with intubation, time on ventilator, time with tracheostomy, comorbidity, and BMI were obtained from the electronic patient records. The Adult Comorbidity Evaluation 27 scale [17], a chart-based validated comorbidity instrument, was used to describe the level of comorbidity in the patient cohort. Data on the respiratory rate during or in connection to the FEES were acquired from the patient records for 23 of the 25 patients.

### Analysis

Videos from the FEES examination were analyzed by one SLP with extensive experience in dysphagia management and one medical doctor specialized in phoniatrics with more than thirty years of experience in the field of deglutition and vocology. Both were certified according the European Society for Swallowing Disorders FEES accreditation program [15]. The ratings of the FEES videos were done together by the two judges in consensus.

The following variables were assessed:

1. Pooling of secretion in the pharynx and larynx before the first swallow was rated using the Murray Secretion Scale (Table 1) [18, 19], a four-grade scale where “1” refers to no or minimal secretion, and “4” to consistent secretion in the laryngeal vestibule.
2. Penetration of bolus and aspiration for all boluses was rated with the Penetration-Aspiration Scale (PAS) (Table 1) applied for FEES [20–22]. The PAS is an eight-point scale were “1” denotes no material entering the airway and “8” indicates silent aspiration to the trachea, i.e., material enters the airway, passes below the vocal fold, and no effort is made to eject.
3. Residue in the vallecula and pyriform sinuses after each bolus was rated according to the Yale Pharyngeal Residue Severity Scale (Table 1) [23–25]. This is a five-grade scale where “1” is defined as no residue and “5” denotes residue filled up to the epiglottic rim or up to the aryepiglottic folds.
4. Recommendations of oral intake rated using the Functional Oral Intake Scale (FOIS) (Table 1) [26]. FOIS is a seven-grade scale where grade 1 is defined as “tube dependent, no oral intake”, and grade 7 indicates “total oral intake with no restrictions”.
5. Movement of the left and right vocal cords (normal, impaired, immobile).
6. Active adduction of the vocal cords during expiration in quiet breathing (“yes” or “no”).
7. Vocal cord lesions (granuloma, bleeding, and/or other; “yes” or “no”).
8. Edema and/or erythema of the left and right vocal folds (none, slight, moderate, severe).
9. Edema and/or erythema of the supraglottal structures (epiglottis, ventricular folds, arytenoid region; none, slight, moderate, severe).

**Table 1:** Rating scales for analysis of FEES examinations.

Murray Secretion Scale [18, 19]	
1	No visible or some transient bubbles of secretion in the vallecula and hypopharynx
2	Deeply pooled secretion in the vallecula and sinus pyriformis
3	Any secretion that changed from a “2” to a “4” rating during the observation
4	Secretion in the laryngeal vestibule
Penetration-Aspiration Scale (PAS) [20, 21]	
1	Material does not enter the airway
2	Material enters the airway, remains above the vocal folds, and is ejected from the airway
3	Material enters the airway, remains above the vocal folds, and is not ejected from the airway
4	Material enters the airway, contacts the vocal folds, and is ejected from the airway.
5	Material enters the airway, contacts the vocal folds, and is not ejected from the airway.
6	Material enters the airway, passes below the vocal folds and is ejected into the larynx or out of the airway
7	Material enters the airway, passes below the vocal folds, and is not ejected from the trachea despite effort
8	Material enters the airway, passes below the vocal folds, and no effect is made to eject.
Yale Pharyngeal Residue Severity Scale [23–25]	
- Vallecula	
1	No residue
2	Trace coating of the mucosa
3	Epiglottic ligament visible
4	Epiglottic ligament covered
5	Filled to the epiglottic rim
- Pyriform sinuses	
1	No residue
2	Trace coating of the mucosa
3	Up to quarter full
4	Up to half full
5	Filled to the aryepiglottic fold
Functional Oral Intake Scale (FOIS) [26]	
1	Tube dependent. No oral intake
2	Tube dependent with minimal/inconsistent oral intake
3	Tube supplements with consistent oral intake
4	Total oral intake of a single consistency
5	Total oral intake of multiple consistencies requiring special preparation
6	Total oral intake with no special preparation, but must avoid specific foods or liquid items
7	Total oral intake with no restrictions

Presence of nasogastric tube for nutrition, tracheotomy (with or without speaking valve), oxygen supply on nasal mask or with high flow nasal cannula during the FEES were noted.

For analysis of proportions of the degree of impairment, ratings from the first FEES were applied in patients who were assessed with FEES more than one time. Possible differences between the first and subsequent FEES examinations were also assessed.

### Statistics

For the main analysis, median, range, and proportions of different ratings of impairment were calculated. Analyses of correlations between ratings of swallowing function and laryngeal function at the time of the first FEES in relation to the total time in hospital, time in the ICU, time with intubation, time on ventilator, and time with tracheostomy were performed using non-parametric Spearman’s Rank correlation tests. The correlation analysis was constrained to variables with data from fifteen patients or more. The rational for this constraint was based on the assumption that the correlation analysis of variables with data from fewer patients was less representative for the whole cohort. In cases of Spearman Rank correlation with a p-value below 0.05, a post-hoc analyses between patients with different rating scores was made using the Kruskal-Wallis test. SPSS Statistics 27.0 for MacOS (IBM) was used for the analysis.

### Ethical considerations

All patients or close family members gave written consent for the patients to participate in the study. The study was approved by the Swedish Ethical Review Authority (approval number 2020–03606).

## Results

Comorbidities of the 25 patients included in the study are listed in Table 2. The time in hospital and the number of days in ICU, on ventilator, with intubation, and with tracheostomy is given in Table 3. Sixty-eight percent (17 out of 25) of the patients were treated in the ICU for more than 21 days. The total time in the hospital was longer than 30 days for 80 percent of the patients. Two patients passed away due to complications from COVID-19.

**Table 2:** Characteristics of patients included in the study.

Characteristics	
Gender, n (%)	
Female	2 (8)
Male	23 (92)
Age, years (mean, range)	63 (43–78)
BMI (kg/m^2^) at hospital admission, n (%)^a^	
Under 18.5	1 (4)
18.6 to 25	3 (13)
26 to 30	11 (48)
31 to 40	6 (26)
40+	2 (9)
BMI at admission (mean, range)	28 (18.4–42.6)
BMI (kg/m^2^) at the time of first FEES, n (%)^a^, ^b^	
Under 18.5	1 (4)
18.6 to 25	9 (39)
26 to 30	7 (30)
31 to 40	5 (22)
40+	1 (4)
BMI at the time of first FEES (mean, range)	25 (16.2–40.3)
Comorbidities, n (%)	
None	6 (24)
Obesity (BMI > 30)	8 (35)^a^
Hypertension	16 (64)
Diabetes	11 (44)
OSAS (Obstructive Sleep Apnea Syndrome)	4 (16)
Stroke	2 (8)
Coronary heart disease	6 (24)
ACE-27, n (%)	
Normal	6 (24)
Mild	9 (36)
Moderate	9 (36)
Severe	1 (4)
Tracheostomy, n (%)	20 (80)
Supine position, n (%)	13 (52)
Kidney dialysis, n (%)	4 (16)

BMI = Body Mass Index. ACE 27 = Adult Comorbidity Evaluation 27 (13).

^a^ Calculated on 23 patients because of missing BMI data for two patients.

^b^ Percentages rounded, therefore does not sum up to 100%

**Table 3:** Total time in hospital and number of days in ICU, on ventilator, with intubation, and with tracheostomy (mean, median, and range).

Criterion	N	Days		
		Mean	Median	Range
Days in hospital	25	57	61	(20–104)
Days in ICU	25	33	31	(6–68)
Days on ventilator	25	25	22	(5–55)
Days with intubation ^a^	25	10	10	(3–21)
Days with tracheostomy ^a^	20 ^b^	30	29	(10–57)

ICU denotes intensive care unit.

^*a*^ Includes total time for patients who were intubated twice or were re-tracheotomized.

^*b*^ Five patients were not tracheotomized

**Fig 1:**
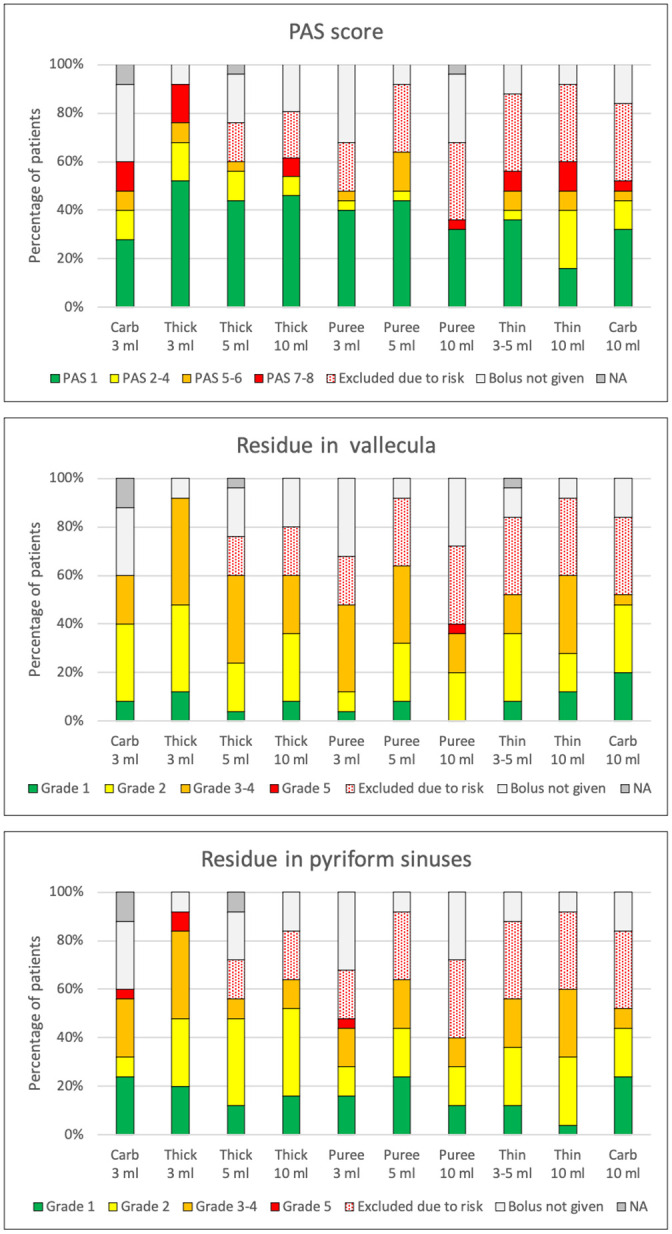
Penetration, aspiration, and residue during swallowing in COVID-19 patients treated in the ICU at the time of the first FEES examination. Proportions of different degrees of impairment for boluses of different consistencies (carb = carbonated water, thick = mildly thick liquid, and thin = thin liquid). PAS denotes Penetration-Aspiration Scale (1 = no penetration or aspiration, 7–8 = aspiration to trachea; see Table 1). Residue after swallow is evaluated with the Yale Pharyngeal Residue Severity Scale (grade 1 = no residue, grade 5 = filled to the epiglottic rim or to the aryepiglottic folds, respectively; see Table 1). Dotted red filling represents patients in whom the boluses were considered unsafe and not given due to high risk for aspiration. Grey filling indicates the proportion of patients who did not receive the particular bolus for other reasons. NA denotes that the rating was not possible to perform due to insufficient visibility on the video.

**Fig 2:**
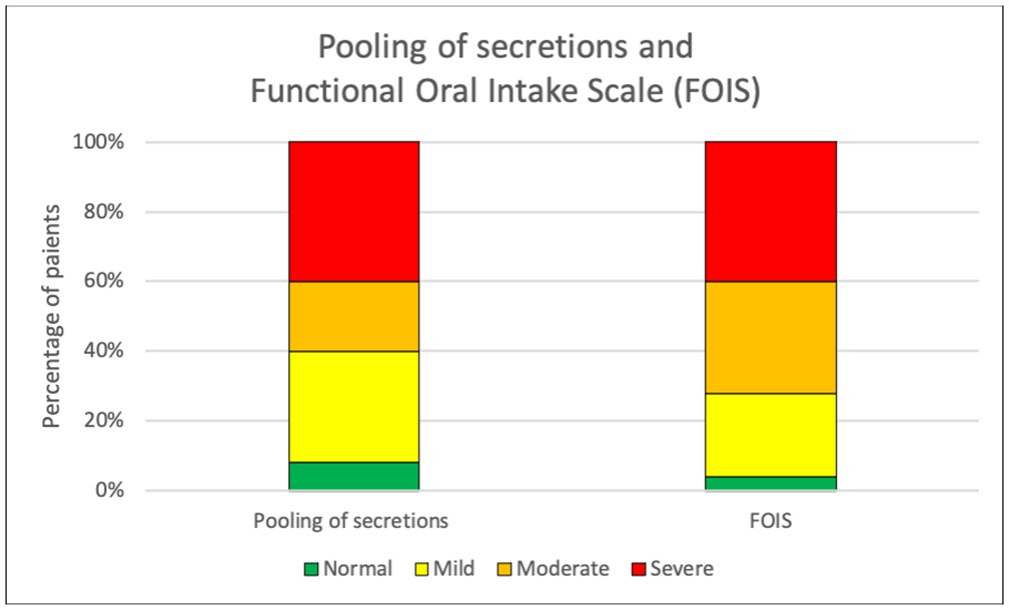
Proportions of pooling of secretions and recommendations of oral intake in COVID-19 patients treated in the ICU at the time of the first FEES examination. Ratings of secretion were made according to the Murray Secretion Scale (“normal” refers to grade 1, no secretion, “mild” to grade 2, deeply pooled secretions, “moderate” to grade 3, transient pooling in the larynx, and “severe” to grade 4, secretions in the laryngeal vestibule). Ratings of recommendations of oral intake were made according to the Functional Oral Intake Scale (FOIS) (“normal” refers to grade 7, total oral intake without restriction, “mild” to grade 5–6, total oral intake with restrictions, “moderate” to grad 3–4, total oral intake with a single consistency or consistent oral intake with tube supplements, and “severe” to grade 1–2, tube dependent with no/minimal/inconsistent oral intake).

**Fig 3:**
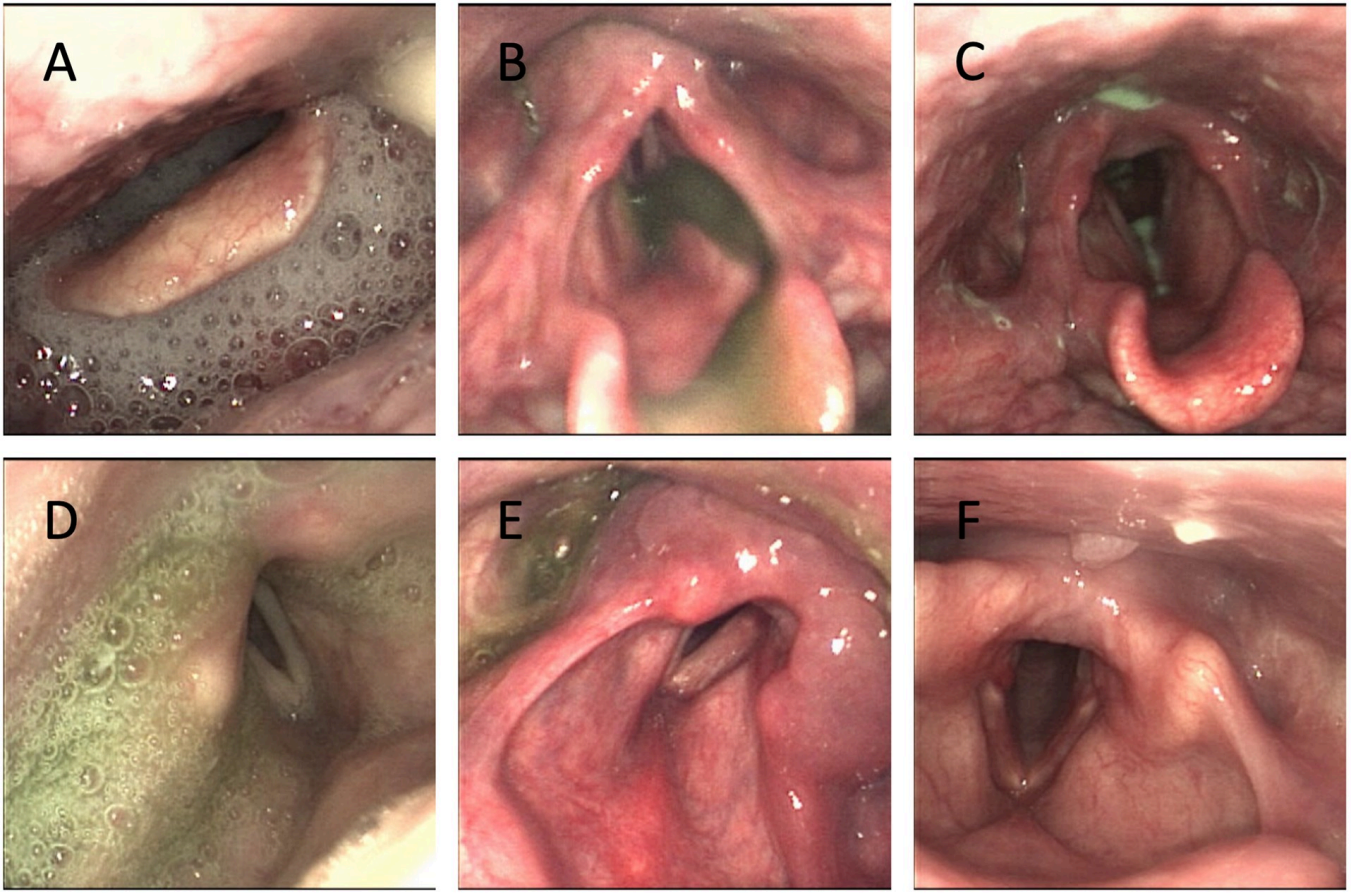
Examples of findings during the FEES. A) Secretions in vallecula and hypopharynx before first bolus. B) Penetration to the level of the vocal folds. C) Aspiration of thin liquid. D) Residue in hypopharynx and larynx after swallow. E) Edema in the arytenoid region on the left side. F) Bilateral vocal fold erythema.

Twenty-four of 25 (96%) patients had some degree of impaired swallowing function with penetration or aspiration at the time of the FEES. There was a variability both between and within patients due to differences in the severity of illness and to the type of bolus given. The proportions of the degree of impairment among the patients according to PAS and ratings of residue, secretion before swallow, and FOIS are presented in Figs 1 and 2. Pooling of secretions before the first bolus was present in all but two patients (92%) in at least one of the FEES examinations (Fig 2). Forty-six percent had consistent secretion in the laryngeal vestibule (grade 4 on the rating scale) and 24% had moderate pooling (grade 3). An example of severe pooling of secretion in the vallecula and the hypopharynx is shown in Fig 3A.

Penetration to the larynx and aspiration was also frequent (Fig 3). Eleven patients (44%) exhibited silent aspiration to the trachea on at least one of the FEES examinations. Aspiration mostly occurred on thin liquid boluses. Fig 3B and 3C illustrates findings of penetration and aspiration.

Residue in the vallecula and hypopharynx was a salient characteristic in a majority of patients, both after intake of thin and thick bolus consistencies (Fig 1). About half of the patients had residue in the vallecula equal to or over grade 3 of 5 according to the Yale Pharyngeal Residue Severity Scale on at least one of the examinations. Slightly fewer had residue of the same degree in the pyriform sinuses. Residue was more common in the vallecula than in the hypopharynx after boluses with puree consistency. Residue in the larynx and hypopharynx after swallowing a small bolus of mildly thick liquid in a patient with severe dysphagia is displayed in Fig 3D.

Twenty-two of 25 patients had enteral nutrition through a nasogastric tube at the time of the first FEES examination. After the swallowing examination, all patients were given at least minimal oral intake as an attempt to regain and preserve swallowing function. Thirteen of 15 patients with serial FEES examinations improved between the first and last FEES (median 11 days; mean 16 days; range 4 to 42 days) and could increase the oral intake. Eight patients could return to full oral nutrition with restrictions and one patient to total oral intake without restrictions. In two patients no improvement was seen, one of whom subsequently received a percutaneous endoscopic gastrotomy tube (PEG) for feeding. Fourteen of 15 patients with several FEES examinations had still some degree of dysphagia at the time of the last FEES. The last FEES examination was often in connection with discharge from the hospital.

The majority of patients had some level of tachypnea during or in connection to the examination. Nineteen of 23 patients (83%) had a respiratory rate over 20 breaths per minute. Of these 19 patients, four had a respiratory rate over 25 and another four a respiratory rate over 30 breaths per minute.

Abnormal laryngeal findings were common (Table 4). Impaired vocal cord movement was found in 76% of patients, including two patients with immobile vocal cords. Only 24% had normal vocal cord movement. A majority (60%) demonstrated with vocal fold erythema, and edema in the arytenoid region in 60% (Table 4; Fig 3E and 3F). Five patients (20%) had focal lesions in the larynx, including two patients with granuloma in the posterior larynx, one with vocal fold hematoma, one with vocal fold ulceration, and one patient with epithelial hyperplasia of the vocal folds (Table 4). Active adduction of the vocal cords at exhalation during rest breathing was noticed in 83% of patients.

**Table 4:** Laryngeal findings.

Laryngeal findings	n (%)
Vocal fold movement	
- Normal	6 (24)
- Left impaired/right normal	5 (20)
- Right impaired/left normal	0 (0)
- Impaired bilaterally	12 (48)
- Left immobile/right impaired	1 (4)
- Right immobile/left normal	1 (4)
Active adduction during expiration	20 (83)^a^
Vocal fold lesions	
- None	19 (80)
- Contact granuloma	2 (8)
- Hematoma	1 (4)
- Ulceration	1 (4)
- Epithelial hyperplasia	1 (4)
Vocal fold edema	
- None	22 (88)
- Slight	2 (8)
- Moderate	1 (4)
Vocal fold erythema	
- None	10 (40)
- Slight	13 (52)
- Moderate	2 (8)
Edema of the epiglottis	
- None	25 (100)
Ventricular fold edema	
- None	23 (92)
- Slight	1 (4)
- Moderate	1 (4)
Edema in the arytenoid region	
- None	10 (40)
- Slight	12 (48)
- Moderate	3 (12)

The results of the correlation analysis are given in Table 5 and S1-S7 Figs in S1 File. In particular, the total time in hospital and days in the ICU appeared to correlate with the severity of dysphagia. The number of days on mechanical ventilation and days with tracheostomy correlated to a lesser degree to ratings of swallowing function. Ratings of vocal fold erythema correlated inversely with total hospital time and arytenoid edema inversely with tracheostomy time. Thus, shorter time in hospital was associated with higher ratings of vocal fold erythema and shorter duration with tracheostomy with more prominent edema in the arytenoid region. There was no correlation between the number of days with endotracheal intubation and swallowing and abnormal laryngeal findings.

**Table 5:** Correlations between FEES ratings and abnormal laryngeal findings vs. duration of treatment for COVID-19.

Variables	Days in hospital			Days in ICU			Days with intubation			Days with tracheostomy		
	N	rho	p	N	rho	p	N	rho	p	N	rho	p
Pooling of secretions	25	0.52 ^a^	0.008	25	0.58 ^a^	0.002	-	-	-	20	0.49	0.032
PAS												
Thick 3 ml	-	-	-	-	-	-	-	-	-	20	0.59	0.010
Puree 5 ml	-	-	-	16	0.52	0.040	-	-	-	-	-	-
Residue in vallecula												
Carb 3–5 ml	15	0.54	0.038	15	0.53	0.044	15	0.57	0.025	-	-	-
Thick 3 ml	23	0.56 ^a^	0.006	-	-	-	-	-	-	-	-	-
Residue in pyriform sinuses												
Carb 3–5 ml	15	0.70	0.004	-	-	-	-	-	-	-	-	-
Thick 3 ml	-	-	-	23	0.44	0.038	-	-	-	20	0.53	0.016
FOIS	25	-0.54	0.006	25	-0.49	0.012	-	-	-	-	-	-
Vocal fold erythema	-	-	-	-	-	-	25	-0.49	0.013	-	-	-
Arytenoid edema	-	-	-	-	-	-	-	-	-	20	-0.61^a^	0.004

Spearman’s Rank correlations. Variables with data from 15 patients or more are included in the analysis. Correlations with a significance level of p <0.05 are shown. Ratings from the first FEES examination were used for the analysis.

^*a*^ Post-hoc Kruskal-Wallis test p <0.05.

**Fig 4:**
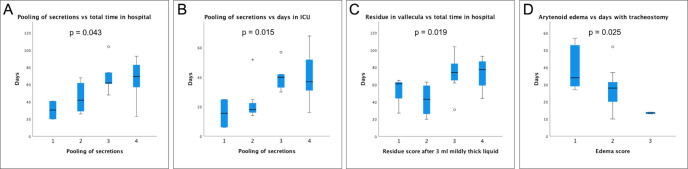
Box plots of FEES variables with a significance of p <0.05 on post-hoc analysis. (A,B) Pooling of secretions; (C) Residue in the vallecula and (D) Arytenoid edema versus total time in hospital (A, C), number of days in ICU (B), and days with tracheostomy (D). Variables with a significance of p <0.05 on post-hoc analysis using the Kruskal-Wallis test are included. Median, 25th and 75th percentiles are shown.

The post-hoc analysis indicates that there was a significant difference between patients with different ratings of pooling of secretions in relation to the total time in hospital (rho = 0.52, p = 0.008; Kruskal-Wallis H = 8.16, p = 0.043; n = 25) and the number of days in the ICU (rho = 0.58, p = 0.002; Kruskal-Wallis H = 10.47, p = 0.015; n = 25), i.e., more severe ratings of secretion were associated with a longer total time in hospital and in the ICU (Fig 4). In addition, there was a difference between degrees of residue in the vallecula after 3 ml of mildly thick liquid with regard to total time in hospital (rho = 0.56, p = 0.006; Kruskal-Wallis H = 9.95, p = 0.019; n = 23) (Fig 4). Patients with low ratings of arytenoid edema differed significantly from patients with higher ratings with respect to the number of days with tracheostomy (rho = -0.61, p = 0.004; Kruskal-Wallis H = 7.39, p = 0.025; n = 20) (Fig 4).

## Discussion

This study describes swallowing function and laryngeal function among 25 critically ill COVID-19 patients treated with mechanical ventilation during the outbreak year 2020. To the authors’ knowledge, this is the first study to evaluate the swallowing function in COVID-19 patients using FEES.

A high prevalence of dysphagia and abnormal laryngeal findings was identified in this series of 25 patients with severe COVID-19 treated in the ICU. All but one had dysphagia to some degree at the time of the FEES either during the later stage of ICU care or after discharge from the ICU. In particular, pooling of secretions in the hypopharynx and residue in the vallecula and pyriform sinuses after swallowing were prevalent. Penetration to the larynx and/or aspiration to the trachea occurred in several patients but was somewhat less frequent. Silent aspiration to the trachea was seen on at least one occasion in 44% of patients. Impaired vocal fold movement, vocal fold erythema, and arytenoid edema were common.

Previous studies have shown that there are several different predictive risk factors for dysphagia and aspiration in ICU patients. Among these risk factors are laryngeal injury caused by the tube during intubation or tracheostomy, neuromuscular weakness, decreased oropharyngeal and laryngeal sensation, impaired cognition and reduced consciousness, impaired coordination between swallowing and breathing, and gastroesophageal reflux. Several factors often occur together [13, 27]. Similar risk factors for COVID-19 patients in the ICU has previously been assumed [28]. Impaired swallowing function in tracheostomized patients could be a result of loss of sensation in the pharynx due to a cuffed tube and/or loss of subglottic pressure with an open tube [29, 30]. Poor coughing ability is seen in both these circumstances, affecting the ability to protect the airway from aspiration.

In this study, a majority of patients presented with residue in the vallecula and hypopharynx as well as secretion pooling. A probable cause for this could be neuromuscular weakness due to long stay in the ICU. Patients with severe pulmonary diseases often exhibit impaired coordination between breathing and swallowing [29]. It can therefore be assumed that COVID-19 patients who suffer from acute respiratory distress syndrome (ARDS) are likely to develop difficulties with breathing-swallowing coordination. Furthermore, most patients were tachypnoeic during the examinations in this study, and it is feasible to assume that a high respiratory rate contributes to difficulties with the coordination between swallowing and breathing. Penetration and aspiration were frequent and probable causes are neuromuscular weakness, injuries in larynx or hypopharynx caused by the endotracheal tube or heavy reflux, discoordination of swallowing and breathing, and poor sensation in the larynx and pharynx [31]. The high incidence of silent aspiration may be due to poor sensation, which can be suspected in many patients. Impaired cognition and awareness were common in these patients and probably had an impact on swallowing function, as seen in previous studies [32, 33].

Dysphagia is a common complication in ICU patients and is associated with adverse events such as higher duration of mechanical ventilation, prolonged hospital stays and a higher hospital mortality rate [13, 28, 34, 35]. Patients suffering from dysphagia are at a greater risk of developing aspiration pneumonitis/pneumonia and malnutrition as well as a later return to oral feeding and impaired quality of life [36–40]. The prevalence of dysphagia in ICU patients varies between studies, likely due to different assessment methods, criteria for dysphagia, and patient characteristics. One study using instrumental assessment, i.e., FEES, found that 58% of patients penetrated or aspirated post-extubation [13]. Another study concluded that 56% of patients aspirated after prolonged intubation and 25% were silent aspirators [41].

Ninety-six percent of the patients in this study had some degree of impaired swallowing function. All of them showed signs of penetration or aspiration. Forty-four percent demonstrated silent aspiration. These are overall higher numbers than in previous studies of dysphagia in critically ill patients. As described, it has been hypothesized that the SARS-CoV-2 virus may induce injury in the central and peripheral nervous system [7], and that this may impact sensory and motor functions related to swallowing [8, 9]. Although we cannot draw any conclusions about the impact of the virus on the sensory and motor functions in this particular patient group, it may be a contributing factor to the ICU-related dysphagia that should be further investigated.

To avoid complications related to dysphagia in this patient group, it seems to be of great importance to evaluate the swallowing function as a standard procedure, preferably at an early stage, before initiation of oral intake. FEES is preferred due to the high incidence of secretion pooling, silent aspiration and residuals. FEES can also be used as a tool to guide decisions for safe decannulation in tracheostomized patients [42]. When discharged from the hospital a large proportion of patients still had some degree of difficulties swallowing and would benefit from a routine follow-up.

Impaired vocal fold movement was found in 76%, and vocal fold erythema and arytenoid edema in 60%, respectively, of this patient group. It is not possible to draw any conclusion from the data in the present study regarding whether this primarily is a consequence of the ICU treatment or related to the COVID-19 infection. Data on laryngeal function after treatment of COVID-19 in the ICU is limited so far. Rouhani et al. [43] reported of findings two months after post-hospital discharge on voice, swallow, and airway outcomes following tracheostomy for COVID-19. Forty-one patients with an average duration of endotracheal intubation of 24 days were included. Eighty-one percent of these patients had normal endoscopic examination of the larynx. Evidence of fixed upper airway obstruction was noted in 22%. More than 50% still had dysphonia after two months, most of them to a mild degree. Signs of laryngeal injury may thus appear to decrease over time in a majority of patients, but adverse effects on the larynx will remain in some. More studies are needed in this regard.

Data from previous studies indicate that laryngeal injury is common after care in the ICU. In a systematic review of the literature Brodsky *et al*. [44] noted a prevalence of 83% of laryngeal injury after endotracheal intubation and mechanical ventilation across nine included studies. The mean time of intubation was eight days. The laryngeal examination was performed within 72 hours after extubation in most studies. Different signs of laryngeal injury were reported. Vocal fold immobility to some degree was present in 21%, vocal fold erythema in 82%, and arytenoid edema in 71% of the total sample of patients. Other findings were vocal fold edema in 70%, granuloma in 31%, and ulceration in 27%. The data in our study suggest that impaired vocal fold movement may be more frequent among patients treated in the ICU due to severe COVID-19 infection, than in the general ICU patient population. The decreased glottal movement may either be caused by arytenoid dislocation after endotracheal intubation [45], or to stiffness of the cricoarytenoid joints due to inflammation secondary to long-term intubation or to the COVID-19 infection. Xu *et al*. [45] also found evidence of neuromuscular damage in some patients with vocal fold immobility after intubation using laryngeal electromyography.

Presence of arytenoid edema was negatively correlated with length of tracheostomy in our study, i.e., edema appeared to be more severe in patients with shorter tracheostomy duration. We have no clear explanation to this somewhat paradoxical finding. It may be that a longer period with tracheostomy reduces the abrasion of the larynx or this may be a coincidental finding. The prevalence of laryngeal injury in ICU patients seems to be related to the duration of endotracheal intubation [44]. In the present study, however, there was no correlation between the number of days with intubation and aberrant laryngeal findings.

A majority of the patients in the present study had visible active adduction of the vocal cords on exhalation during tidal breathing. Expiratory glottal narrowing can be found in, for example, individuals with asthma [46] and chronic obstructive pulmonary disease (COPD) [47]. A slight glottal narrowing during expiration is also seen in some healthy individuals during breathing at rest [48]. There appear to be a coupling between the pattern of glottic movement and the respiratory pattern [49]. For example, glottal adduction is enhanced during loaded expiration [50]. The majority of patients in our study had some degree of tachypnea. The active expiratory movement of the vocal folds may be related to an increased respiratory workload due to respiratory insufficiency. It may also be a means to increase the intrinsic post end-expiratory pressure (PEEP), as has been proposed for COPD [47]. Further studies are needed to clarify this.

Even if the number of patients in this study is greater than in previous studies investigating dysphagia in COVID-19 patients using FEES, a limitation of this study is the small sample size. Another limitation is that the data is not representative for the entire COVID-19 patient population due to the selected sample. All patients were referred on initiative of the ICU/ward and it is likely that the patients who were referred were the ones with greatest difficulties. While most patients had a high degree of dysphagia, there was a great variation among patients within the study group in terms of morbidity and degree of dysphagia, making it impossible to draw any conclusions about the entire population of COVID-19 patients.

The results from this study highlights the importance of evaluating dysphagia and laryngeal findings in COVID-19 patients at an early stage to prevent dysphagia-related complications and to offer adequate intervention and safe return to oral feeding. Further research with larger study populations is warranted, as well as consistent follow-ups over time for this particular patient group. A study of patients with COVID-19 treated in the ICU with regards to swallowing function in the short- and long-term is ongoing at our hospital.

## Supporting information

S1 File (PDF)
