# Supplementary material for: Characterization of dysphagia and laryngeal findings in COVID-19 patients treated in the ICU—An observational clinical study
Source: PLoS One. 2021 Jun 4;16(6):e0252347. doi: 10.1371/journal.pone.0252347 (PMC8177545; doi:10.1371/journal.pone.0252347)
Supplement: S1 File — (PDF) [file pone.0252347.s001.pdf]

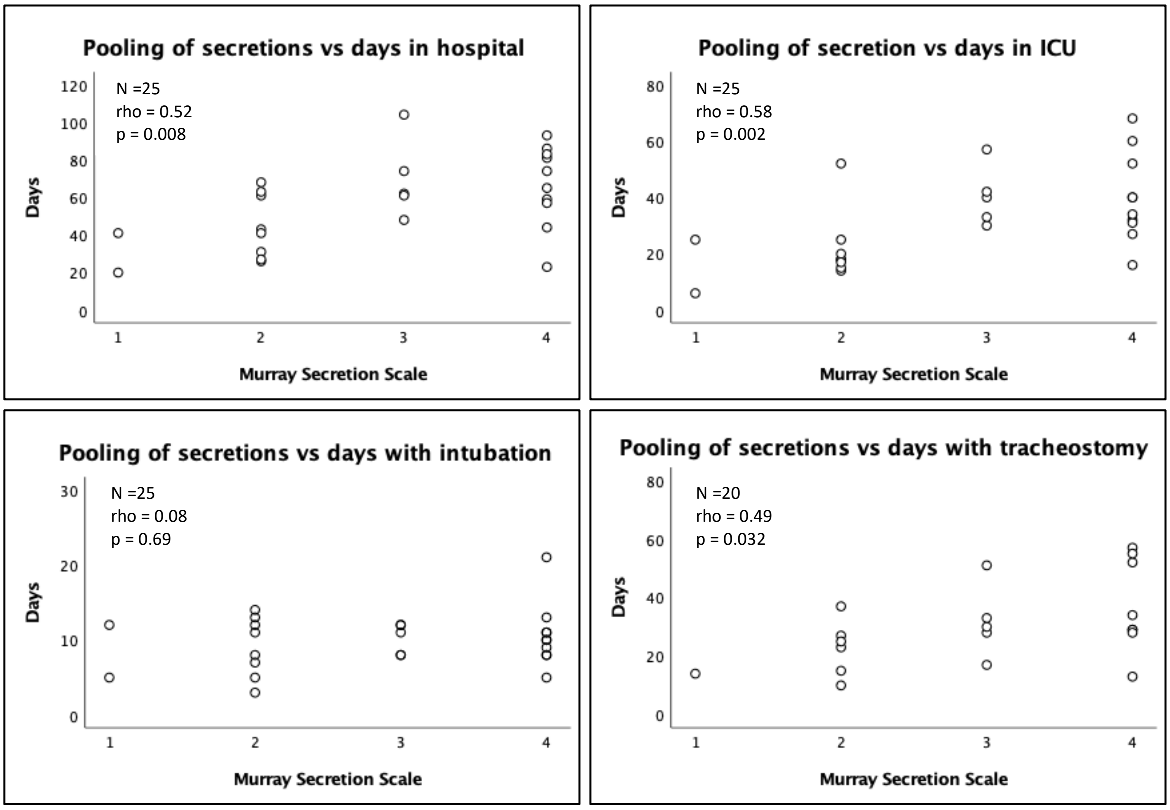

S1 Fig. Scattergrams of ratings of pooling of secretion before first bolus vs duration of care

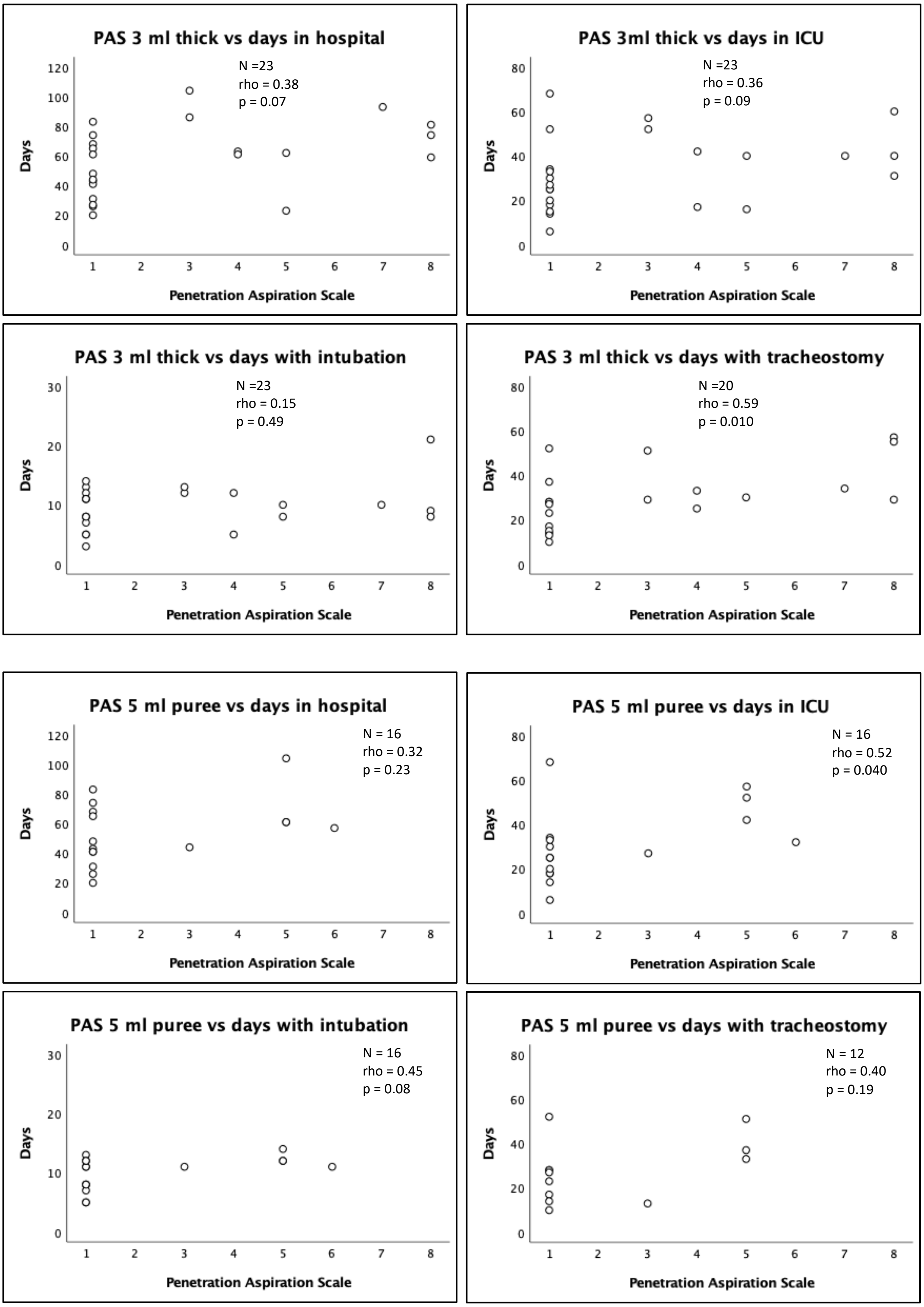

S2 Fig. Scattergrams of rating of Penetration Aspiration Scale (PAS) vs duration of care

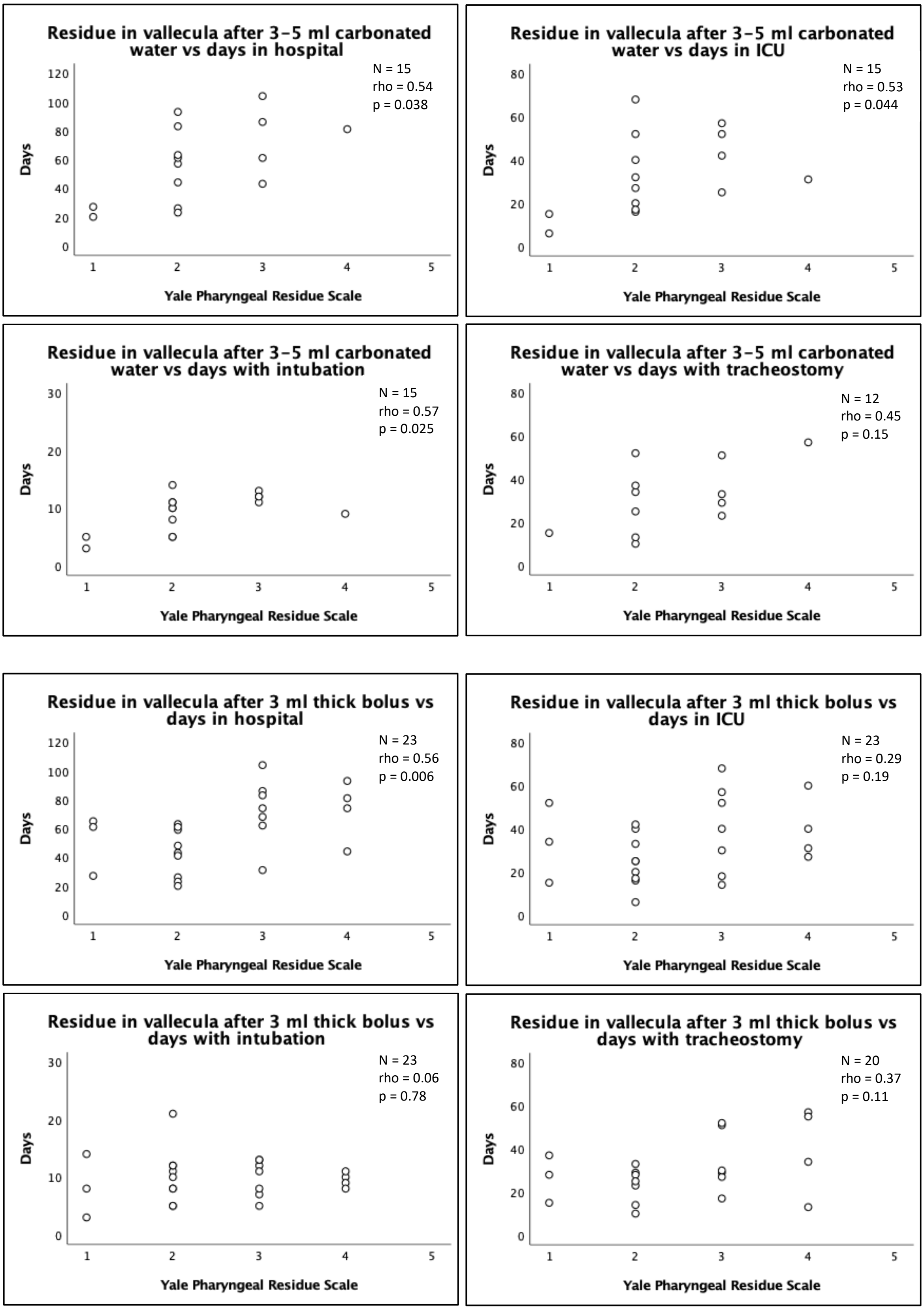

S3 Fig. Scattergrams of ratings of residue in the vallecula vs duration of care

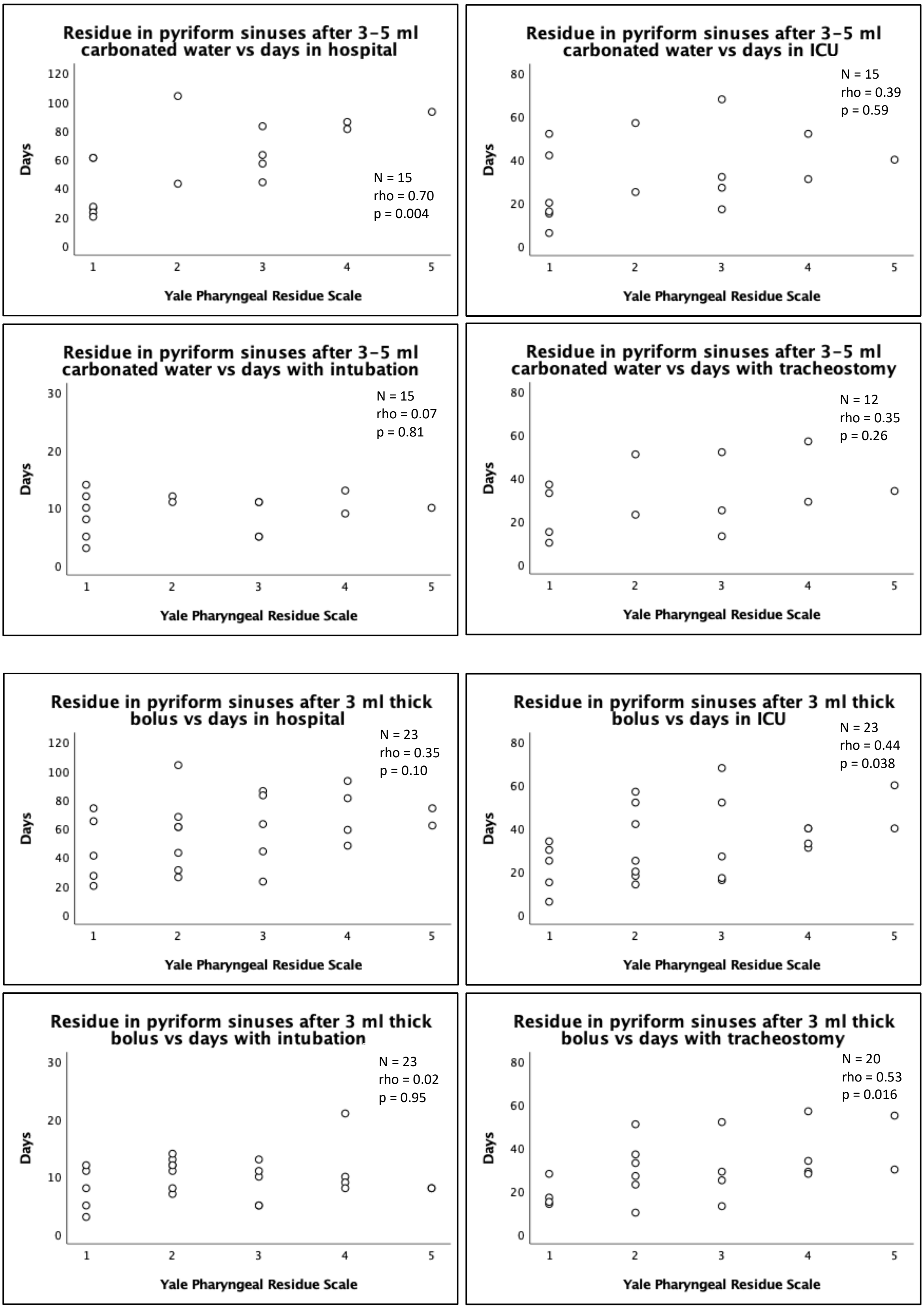

S4 Fig. Scattergrams of ratings of residue in the pyriform sinuses vs duration of care

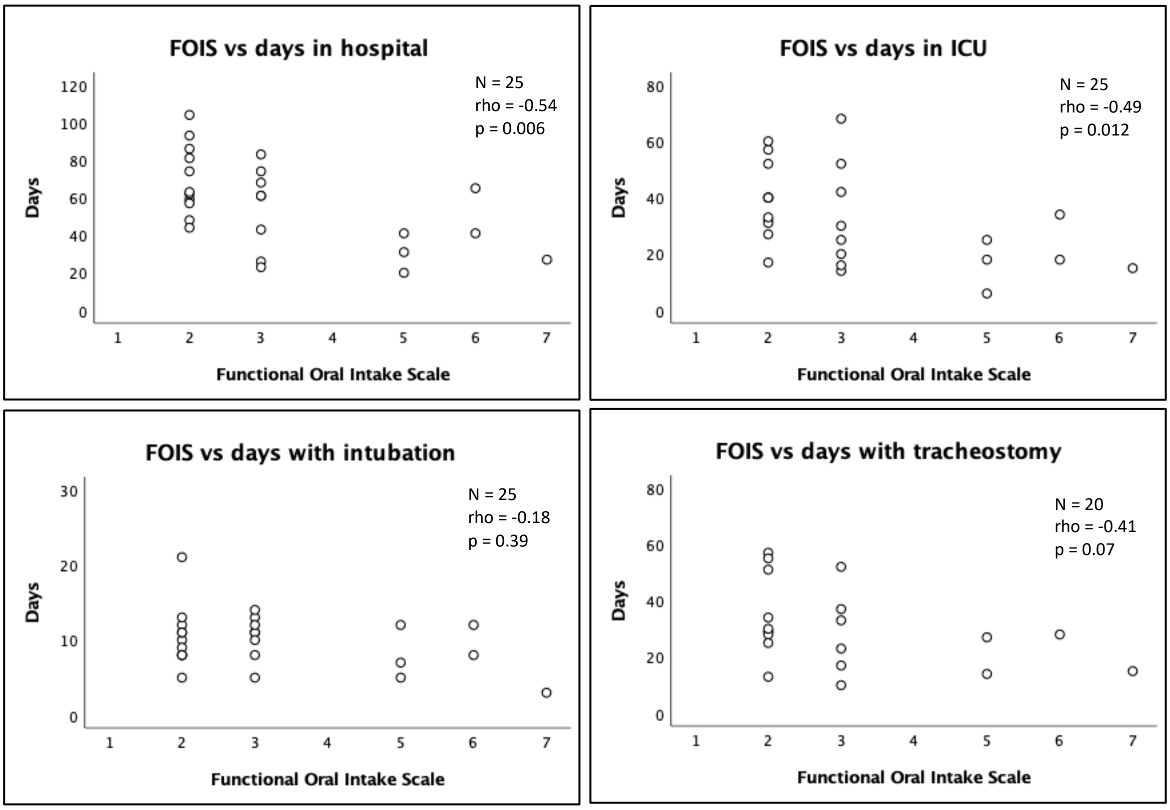

S5 Fig. Scattergram of Functional Oral Intake Scale (FOIS) vs duration of care

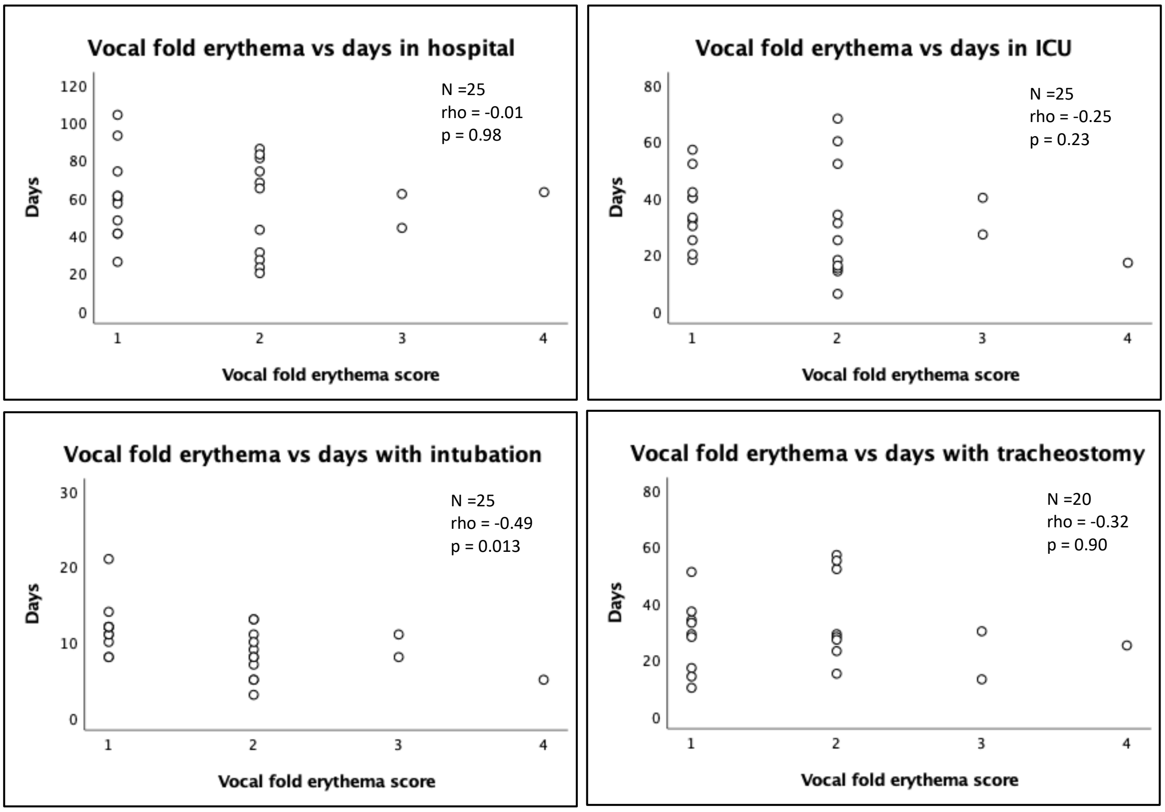

S6 Fig. Scattergrams of ratings of vocal fold erythema vs duration of care

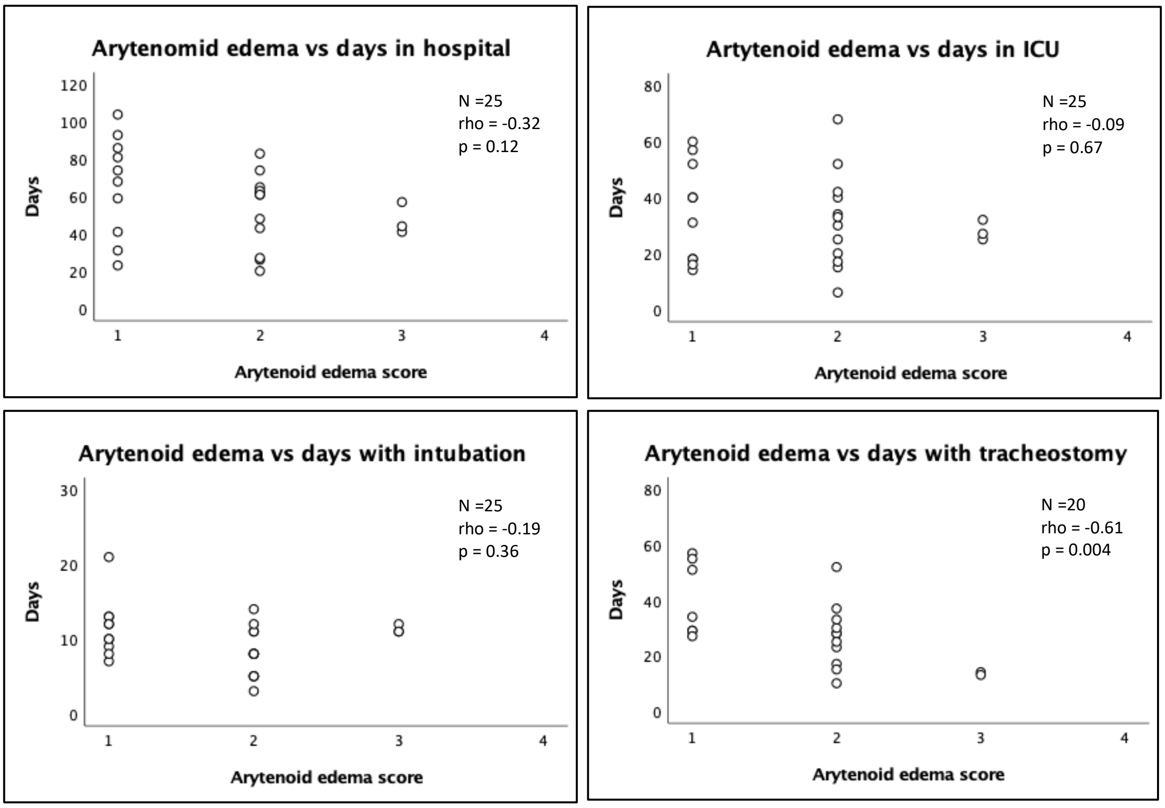

S7 Fig. Scattergrams of ratings of arytenoid edema vs duration of care
